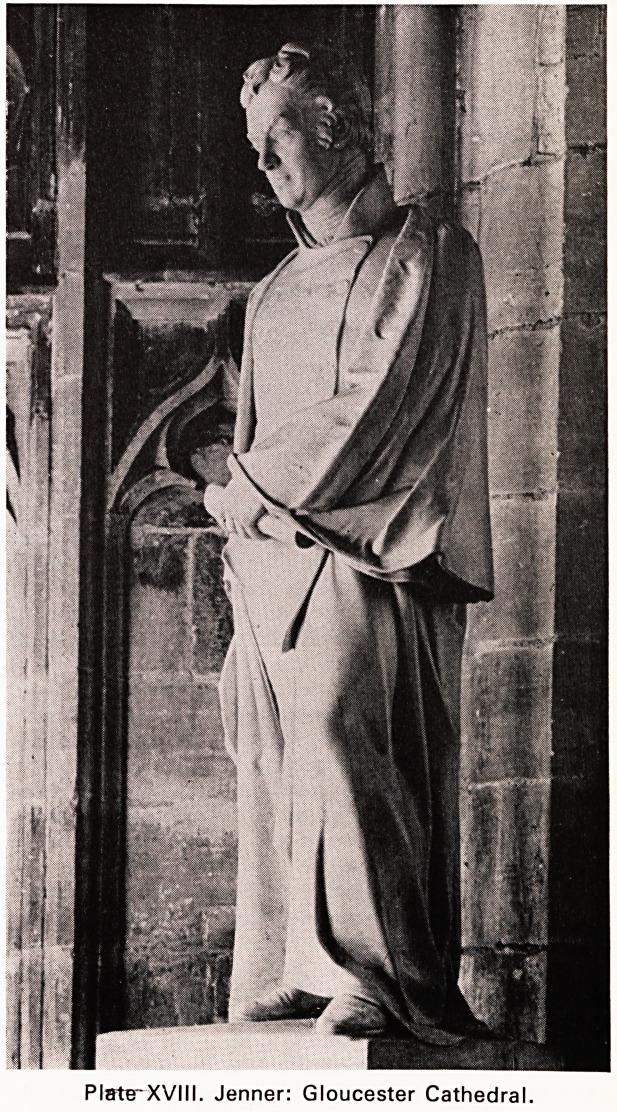# Edward Jenner—A Great Englishman
*Presidential address to Bristol Medico-Chirurgical Society, October 1972.


**Published:** 1973-04

**Authors:** James Macrae


### Edward Jenner—a great Englishman*

**James Macrae**

Ladies and gentlemen, I cannot express adequately my feelings of appreciation at the unexpected honour you have paid me in electing me President of this old, respected and vital Learned Society. I thank you with all the sincerity at my command and have accepted the task with humility and in the hope that I may serve. You know me to be a Celt with the implied limitations. I must confess to deficiencies, both congenital and acquired, and thus sit here before you in the shadow of these.

Before I approach the subject proper of my talk, I wish to have some help and company beside me: I wish to try an experiment in time. Time is one of the words we use constantly and glibly and yet none of us know its real meaning. In 1923 my maternal grandmother, born in 1840, spoke to me of her childhood in Lochgilphead. Her father talked to her about how he visited Bristol as a seaman and how he heard there of a great doctor who lived nearby, Dr. Edward Jenner. Two voices from the past made me aware of the man who is now by my side in spirit.

Fortified by this timely support, I suggest to you that today is the 17th May, 1749, and the vicar of Berkeley, Gloucestershire, has just celebrated the birth of his eighth child, to be christened Edward by himself. The child's mother died in childbirth in October 1754, followed by his father in December of the same year. Young Edward was left in the care of his eldest brother, a young clergyman of twenty-two, newly fledged from Oxford, and a married sister living in Berkeley.

Educated locally at Wotton-under-Edge and Cirencester, Edward Jenner became apprenticed to Daniel Ludlow, a surgeon of Sodbury at the age of thirteen. Seven years of this customary training in medicine was capped by the unusual experience of being the first resident pupil of John Hunter in the new St. George's Hospital, London. This vastly improved his medical education and made him a life-long friend of that mercurial, if irascible, Scot. From his youth he was observant and inordinately interested in the lives of everything about him. He was a natural naturalist trained by his own curiosity. Obviously, he regarded Hunter as his hero, mentor and friend. Hunter made use of his assiduous young disciple: corresponded regularly for twenty years and issued orders which Jenner obeyed implicitly trying to keep pace with the erratic genius of Hunter's superabundance of ideas.

Plate XV. Edward Jenner, aet. 52

### Jenner and Hunter

The times were exciting, with the birth of the United States: the French Revolution was brewing and exploded: simultaneously, a strange stirring of intellect was evident in the persons of Linnaeus, Gilbert White and Captain Cook to mention only a random sample. Jenner, a well qualified doctor and a naturalist was, according to report, a great help to Banks, the naturalist to Cook's first voyage. He was acknowledged by Banks and offered the post of naturalist on the projected second voyage. Fortune awaited him as a fashionable physician in London and fame in the ends of the world as a naturalist. He chose quite firmly to be a family doctor in Berkeley, a task which occupied the major part of his remaining life. Thus, about 1772, he made a great, independent and surprising decision. It was no mistake, suiting the man, his personality and his proclivities, better than the material laurels offered in the metropolis. He left his undoubted hero, John Hunter, but appeared to be very content to use the pen as a bridge: perhaps a small price to pay for the absence of Hunterian bad temper!

Jenner achieved that difficult end, confidence and trust, from the folk who knew him as a child. He became the beloved physician in a wide area, quickly and on his merits. He was a quiet leader, establishing and maintaining two medical clubs for his colleagues, did his own post mortem examinations, never failed to visit anyone who called for help and kept notes about everything. These were accurate. Nature sur-

*Presidential address to Bristol Medico-Chirurgical Society, October 1972.

rounded him, life moved at horse-speed, not horse-power, so he never failed to note the behaviour of all things, quick, dead or abstract: he remembered for his own pleasure or of anyone who accompanied him. He wrote to Hunter who in turn issued orders. Hunter was a genius but a technician, careless of himself as well as others: he wanted to know, at once. Jenner was a man, caring for all things, even himself, and he was slow to change his course — the very antithesis of Hunter.

Plate XVI

Jenner wrote of his love for a young woman, Hunter brushed such triviality aside with a remark that all women were alike and scolded Jenner for not making proper reply to a former enquiry. Jenner bore the older man's graceless language with calm and obeyed with skill and endless endeavour, whenever possible. Hunter destroyed, or lost Jenner's letters, Jenner treasured those of Hunter so that we can, today, read them in the Royal College of Surgeons. Hunter went to Oxford for a few weeks to obtain a medical qualification which he lacked and dismissed the city and all its works as not worthy of his attention. Jenner distrusted his own knowledge of Latin so firmly that he refused to try the Latin examination set by the Royal College of Physicians. So, the two friends for over twenty years lived, until 1793 when Hunter died, almost at his own hands, the result of a bad experiment his quick mind determined on years before.

In 1783, Hunter suggested that the habits of the cuckoo might be worth investigating: so in 1787 after much tedious work Jenner reported an odd story to the Royal Society. He was not believed and told to look again. He transmitted the same tale in 1788 by which time his careful observations had been checked sufficiently to warrant guarded publication in the Philosophical Transactions and he was admitted a Fellow of the Royal Society. Just recently, present day photography has vindicated everything Jenner observed and reported.

Hunter sent him a home-made thermometer to take the rectal temperatures of hibernating hedgehogs— Jenner did as he was bid. Hunter accepted the result but commented that it would have been better to make a small abdominal incision to admit the thermometer! Neither Jenner nor the hedgehogs took any heed of the outrageous idea. Such were samples of the multifarious duties Jenner undertook for his task-master while he enjoyed himself as doctor and naturalist.

His medical notes were no less meticulous: he noted the state of the coronary arteries in patients who had had angina pectoris and prescribed a mercurial ointment of his own devising for ophthalmia: he recorded his own symptoms and signs during attacks of typhus and frostbite and in 1792 two medical friends recommended him to their own university of St. Andrews, and his medical skill earned him the Doctorate of Medicine in the oldest university in Britain.

**Vaccination**

For at least ten years he mulled over the well-known country tale that milk-maids did not get small-pox if they had had cow-pox. He started to collect details from dairymaids and cattle men but the investigation was slow because cow-pox was not common. He wrote to Hunter, but the great man was not very interested and of course died in 1793 so that Jenner was alone with his problem. Small-pox was a plague, mostly among children, with a high mortality and an unimaginable morbidity. Few of the large families of the day escaped, even Royalty.

After these ten years of thought, Jenner believed he held the complete prevention of small-pox within his grasp and he clung to that belief until his death. He waited for cow-pox: it came with a cow named 'Blossom' and a dairy-maid, Sarah Nelmes, who presented with well-nigh perfect cow-pox lesions on her hand. Jenner inoculated an eight year old boy, James Phipps of Berkeley, on 14th May, 1796 with matter from Sarah Nelmes' lesions. James had not had small-pox: on 1st July he was re-inoculated with real small-pox and the disease refused to take. Jenner was overjoyed, he gave young Phipps a small house near the church tower and that same tower has "J. PHIPPS— 1799" carved in the stone—perhaps the work of the same boy.

Jenner could not resist sending his small body of evidence to Sir Joseph Banks, now President of the

Plate XVII. View from the Chantry garden

Royal Society, who was not impressed, but kept the documents. Undeterred, Jenner inoculated more and more people, using the fluid from one person's lesions to inoculate others thus rendering the cow an unnecessary intermediary. He even stored some of this material in the quills of hens' feathers and sent these to his medical friends. Soon his fame spread beyond Berkeley and patients inoculated, or "vaccinated" by Jenner did not get small-pox. In 1798, he published privately an account of his studies and results. He went to London to spread the good news. His naive good nature was surprised by the indifference of his medical colleagues and he left London for Berkeley very disappointed. One of his rustic hen's quills was left in the hands of Henry Cline, who quite by accident found Jenner to be right. Thus started a doctors' war against Jenner, waged largely by Drs. Woodville and Pearson who tried by all means to discredit him and caused the country physician much pain and no little loss of confidence in the ethics of his profession.

His thesis was widely read abroad and the worth of his discovery was acclaimed everywhere, even by the arch-enemy, Napoleon. The British Government grudgingly acknowledged the Gloucestershire doctor and granted him £20,000 in dribs and drabs as governments do. Jenner spent most of it on furthering the case of Vaccination. He was still busy as a country doctor, married, with a family and much to do and yet he was further tormented by a cult of Antivaccinationists with an active branch in Gloucester city much to our doctor's dismay. These people's fanciful ideas about Jenner's work took the clever caricaturists of the time away from a diet of Pitt, the Wars and 'Boney' and they revelled in depicting Jenner as an ogre who caused babies to grow cow horns — and worse. Jenner was very hurt indeed. All this at home, while the world showered him with honours, from the Empress of Russia to the Red Indians of America: he remarked, in a letter that honours 'buy no mutton'. By 1801, he had published his "Inquiry" in three editions. In 1970 a first edition copy was sold for nearly £1,000 in Bath.

In appearance, Jenner was not unlike any of us: he did not wear a wig, possibly his naturalist hobbies precluded this: he was at home with all sorts of people: he was happily married. Something of a poet, his verse spoke of the country he loved; he had his troubles when his eldest son died and his wife followed in 1815. He was sometimes irritable and depressed, he was religious with the passion of someone who knows man's intellect is limited and there must be a Higher Being, the Author of the Universe, and to be glorified. He enjoyed good food, singing, played the flute and fiddle and he wrote many letters although he was not literary. He joined freemasonry and after his death from a stroke in 1823, many masons from far afield held a memorial service in Gloucester Cathedral, giving sufficient money to have a magnificent statue (Plate XVIII) erected in 1825 to his memory beside the West Door. He lies at home with his family in Berkeley Church and his vines still bear fruit in the Chantry garden.

~~Plate~~ XVIII. Jenner: Gloucester Cathedral.

**Greatness**

I have lived among you since 1946. Six years with the Army, some five of these years abroad in many countries, under the protective umbrella of British bayonets at war, broadened my outlook but I have remained a Celt, apart, and yet living in England. I am at best a technician like Hunter and my hobbies have been few. However, all this has allowed me to stand back and observe the reactions, interests and workings of the English for a quarter of a century. I have always been intrigued with history and with words. My history has not been academic but rather peripatetic and words must have exact meanings to please me. My favourite historians have been men like Arthur Bryant and John Prebble who make their characters live and the "Kings and Queens" type of history has been meaningless. Possibly Edward I was the only Royal figure with greatness in his make-up: yet his son Edward II lies as a Saint in Gloucester Cathedral after an inglorious life ending in a peculiarly tortured death in Berkeley Castle at the hands of two gentlemen from Bristol. To be canonized in Gloucester was an example of the English way of putting things right. The Celt never forgets insult.

When the Romans came to Britain they found Celtic tribes, ununited and quarrelsome with noble figures, like Caractacus and Boadicia. They found Druids, Picts, Scots from Ireland. They found no great men. The Roman troops were Europeans not citizens of Rome and so started the first recorded invasion of these islands. Roman soldiers stayed, occupying the best land cutting down much timber and pushing westwards the native Celt as has happened along the seaboard of Europe from Spain to Scotland. The only area not involved overmuch was that troublesome island, Ireland.

After the Romans came successive invasions by Angles, Saxons, Jutes, Scandinavians and indeed all the Europeans pushed west by the Mongol pressure from the very far East. Thus began the mixture of race and culture which has continued to this day. The so called Norman Conquest brought the order and discipline of the Feudal System but it was no conquest in the real sense and the English absorbed the French-Normans like all the others, then and since.

The island isolation allowed gradual incursions of Germans, Huguenots, Flemings, all with some gift to contribute. A century ago the Jews from Russia and latterly Germany contributed more than is realised. Always new blood has been added to a population with a basic love of the land. Such a genetic whirlpool is very different from the useless idealism of the Celtic twilight lost in dreams and inbred to extinction. Incredibly, this genetic sewer has contributed greater men to the human race than any other nation on earth. There have been mistakes, wars and horrible incidents, as the nation grew up but always great men appeared, not from the aristocracy, so much prized by the pedigree conscious Celt, but literally from every layer in society. Tolerance and welcome for strangers has always been there and innate stubbornness has never allowed admission of defeat. Today we are witnessing possibly the greatest experiment of all, in the absorption of coloured people into the society. It will be a great success in the future and the English will benefit as they always have done.

And here is where I come to my second hobby—words. What makes a great man? Is it due to the gift of brains, genius, brawn, cruelty, saintliness or any of the attributes so often associated with fame? England has never lacked men with these gifts: poetry, literature, inventiveness, administrative abilities, politics, games, religions and luck have appeared here first often and in plenty. The English gathered an immense Empire by accident and gave it away. The Celts, Scots, Welsh, Irish and Cornish, have helped, lending verve, élan, technical ability and have made Good Empire Builders but they have proved inconstant by clinging to a nationalism becoming progressively less important; refusing to admit that ancient languages are unimportant and that English is likely to become the world's answer to the problem of Babel. Scotland exports education, Wales technology as it did at Agincourt, and Ireland labour and revolution. England absorbs and remains slow to anger but terrible when aroused at last. England loves to say it will muddle through but in reality the men for the job are always available backed up by the resolution and steadiness of purpose of a whole nation. It was this extraordinary steadiness which brought Moore's carefully trained Light Divisions from Folkestone through the terrible privations of the retreat to Corunna and helped Wellesley win the well-nigh impossible battles at Talavera and Badajoz, just as the same men wrought wonders later at Dunkirk and Alamein. Nelson's pressganged sailors never failed him nor did he expect failure. And yet in these perilous days life went on at home without panic or even realisation of the dangers afoot. It is this quality, I believe which has made the English nation great, combining tolerance and careful indiscretion in moments of crisis, relapsing into apparent apathy when the danger is past.

Such were the men who surrounded Jenner, plodding on about their own business but with a goal clearly to be reached however far distant. They are to be seen today, astute, lovable countrymen, but immovable in their chosen ways.

Thus I propose that Edward Jenner was greater than John Hunter: just as the young man of the Gloucesters, fighting in Ulster today, is greater than any idealist I.R.A. man behind a murderer's gun.

Greatness is a constant quality of the whole man for a life time, not for a moment. The land of England produces such men and perhaps it is a quality of the land not of genetics. Of course, there are poor products of every potter's wheel and count for nothing but are cast aside; the proportion of great men in England has been large and they have all been men in the truest sense of the word unconscious of their greatness.

**A memorial**

As usual the prophet has little honour in his own country. So it has been with Edward Jenner. There was no local institution in his honour in Berkeley, or elsewhere, until 1967 and my personal inquiries among medical students during those many years I taught in Bristol, have been little rewarded. Some had heard of him, few knew he lived at Berkeley only sixteen miles away and I never met one who had visited the place.

The establishment of the Jenner Trust was the achievement of one man, who should speak here in my stead, but may not—Dr. Malcolm Campbell. To him alone belongs the initiative and drive which started in Berkeley churchyard in October 1965 and culminated in the opening of the Jenner Museum in James Phipps' cottage, and the legal establishment of the Jenner Trust in 1967; a great task involving constant endeavour, now in our hands to be maintained as Malcolm Campbell intended, for posterity.

Why should John Hunter be remembered so adequately in London? William Harvey's portrait appears beside Jenner's portrait in Berkeley Castle. Harvey is commemorated by an oration in London and both Hunter and Harvey carry honorary professorial titles for their orators.

May I suggest Bristol does the same for Jenner, the greatest of the three? And let the lecturers be young people: Jenner gives a wonderful choice for young surgeons, physicians, general practitioners, naturalists or veterinary surgeons. He was all of these, and many more.

I have listened to every Presidential Address since 1947. Our Secretary reminds me that in that year Dr. Milling spoke of Jenner and I am glad to follow his example in 1972, two hundred years after Jenner took that most important decision to return from London to Berkeley. Bristol owes him something for that decision.

Each President has taken the job seriously, as I find I must, and in his Address showed some facet of his or her character hitherto unsuspected by me: pernaps an interesting observation. I hope I have not been too transparent.

Thank you for listening.

## Figures and Tables

**Plate XV. f1:**
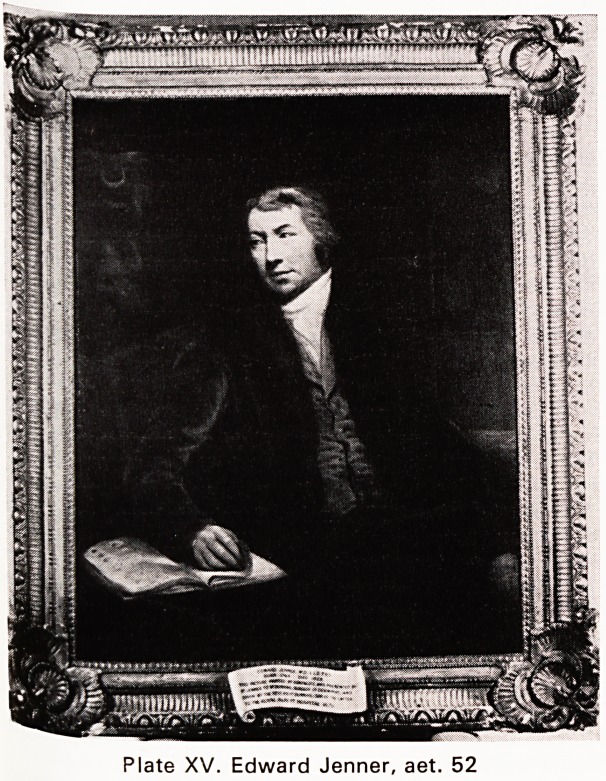


**Plate XVI f2:**
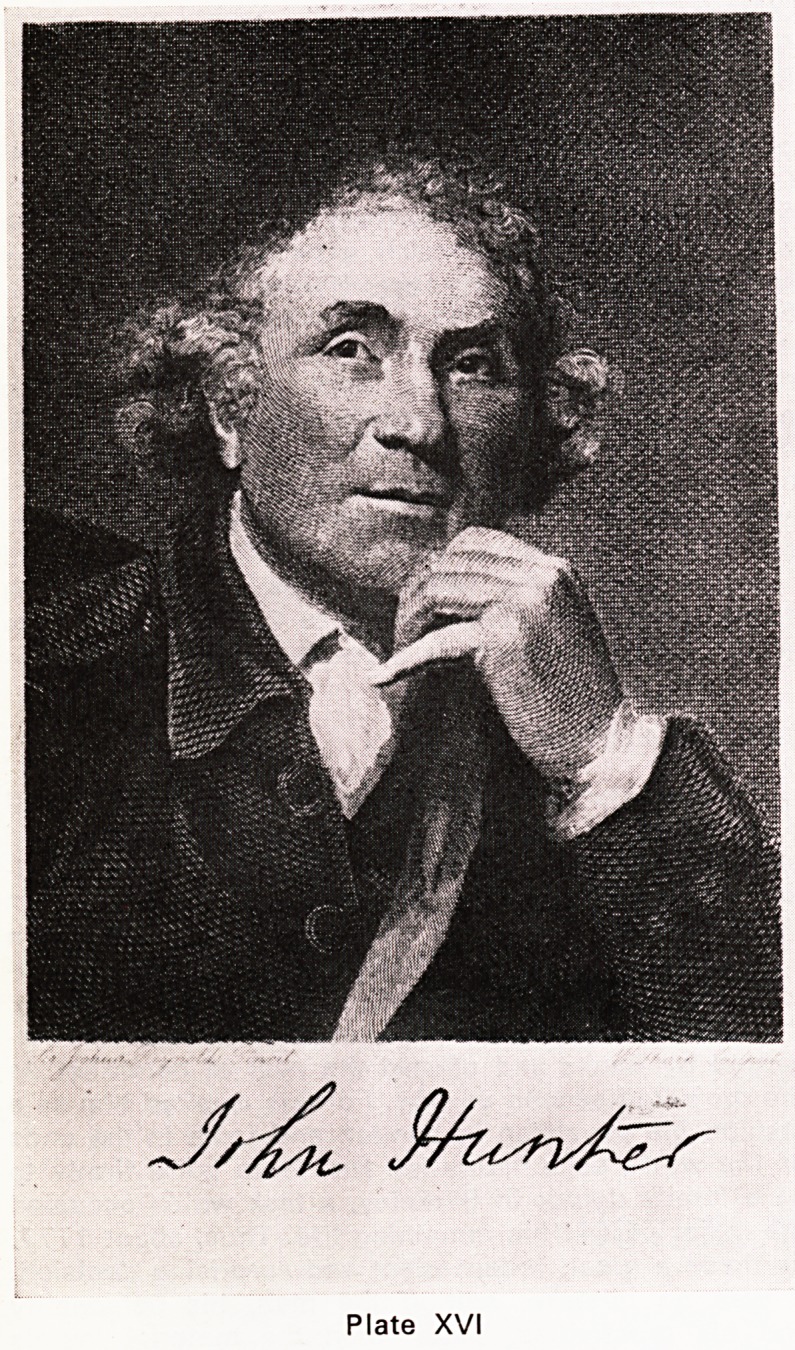


**Plate XVII. f3:**
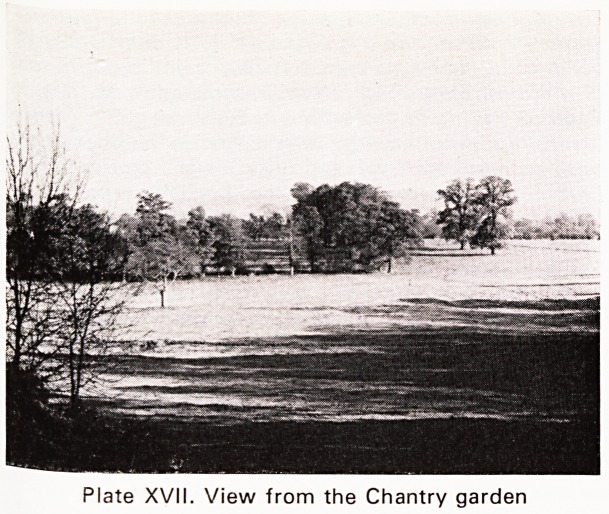


**Plate XVIII. f4:**